# Referrals from general practice to consultants in Germany: If the GP is the initiator, patients' experiences are more positive

**DOI:** 10.1186/1472-6963-6-5

**Published:** 2006-01-19

**Authors:** Thomas Rosemann, Michel Wensing, Gernot Rueter, Joachim Szecsenyi

**Affiliations:** 1 Department of General Practice and Health Services Research, University of Heidelberg, Voßstr. 2, 69115 Heidelberg, Germany; 2 Centre for Quality of Care Research, Radboud University Medical Centre Nijmegen, P.O. Box 9101, 6500 HB Nijmegen, The Netherlands; 3 GP network Marbach, Blumenstr. 11, 71726 Benningen, Germany

## Abstract

**Background:**

Referrals of patients from primary care to medical specialist care are an important activity in any health care system. German data show that the number of referrals by GPs have increased since 2004, but detailed insight into the experiences of patients, GPs and consultants regarding referrals is very limited. This study aimed at describing the experiences of consultants, GPs and patients with referrals from primary care to medical specialist care. An additional objective was to examine the impact of purpose regarding the referral and of the referrer on the experiences of GPs and patients.

**Methods:**

Referrals of 26 general practitioners (GPs) from 25 practices in Marbach, a rural region in the south of Germany were studied. All adult patients referred after consulting these GPs in a period of five weeks were eligable for the study. GPs, consultants and patients completed short structured forms to document factual characteristics of each referral and their experiences with the referral. GPs and patients completed forms before and after the referral was made, while the consultants completed forms after the patient had consulted them.

**Results:**

Overall, consultants were very positive about appropriateness of the referral (91%). They were somewhat more critical regarding the information provided on the patients' medical history (61%) and prescriptions (48%).

In 258 referrals (63%) GPs perceived clear diagnostic benefits, while in 202 referrals (49%) they perceived clear treatment benefits. GPs' experiences were more positive if the GP's purpose was to reduce diagnostic uncertainty (beta = 0.318, p < 0.001) or if the purpose was to exclude serious illness (beta = 0.143, p < 0.010). Other purposes of the referral had no impact on their experiences.

Patients' expectations regarding the referrals mostly referred to diagnosis, including increased diagnostic certainty (80%), detailed information about the illness (66%) and exclusion of serious illness (62%). They were overall satisfied with the referral (83%). Their experiences with the referral were more positive if the initiative for the referral came from the physician (beta = 0.365, p < 0.000).

**Conclusion:**

Patients, GPs and consultants have positive views on the value of referrals from primary care to medical specialists. Patients were most positive if the physician had initiated the referral, which supports the gate keeper role of the GP.

## Background

Referrals of patients from primary care to medical specialist care and back to primary care, comprise an important activity in any healthcare system [1]. Optimal referring processes are crucial for the effectiveness, safety and efficiency of medical care [2]. Across Europe delivering and financing care in the primary care sector is quite differently organized. There is evidence, that the gate keeping role of general practitioners (GPs) increases efficacy of the system and reduces costs [3]. Due to decreasing financing resources and the potential effectiveness and efficiency of a primary care centred system, an intense political discussion has started on the gate keeper role of the GP for access to hospital care in Germany [4]. Since 2004, patients can access medical specialists directly, but as a first step towards a GP gate keeper role they have to pay an additional fee if they do not consult their GP first. Moreover, the GP can determine if the specialist is allowed to refer the patient to other medical specialists or if he has to be referred back after the consultation. National data showed that due to the introduction of this fee, the number of referrals from primary care to hospital care has increased tremendously. Consequently, the quality of communication between GP an specialists has come into the focus of health care professionals and politicians. Critics argue for example, that it may cause dissatisfaction among patients that the GP has to – at least formally – initiate the referral. However, data on the actual experiences of German patients, GPs and consultants regarding referrals were hardly available.

An optimal referral has a clear purpose, related to diagnosis or treatment, which is specified by the GP in the communication with the consultant. Also, patients have specific expectations of the referral, related to diagnosis or treatment, which may or may not have been discussed with the GP. We wondered, whether the GPs' and the patients' experiences were associated with the purpose or expectation regarding the referral. We hypothesised, tentatively, that diagnostic goals would be clearer and more feasible to accomplish, resulting in more positive experiences in GPs and patients. Furthermore, we expected that patients' experiences would be most positive if the referral was initiated by the patient.

## Methods

### Design

A prospective observational study was performed in June and July 2004. The ethics committee of the University of Heidelberg gave approval for the study. All patients gave their written and informed consent.

### Sample

Referrals of 25 general practitioners (GPs) from 25 practices in Marbach, a rural region in the south of Germany were studied. All adult patients referred to consultants by these GPs in a period of five weeks were eligable for this study if they were personally seen by the GP. In the first week, all referrals on Monday were included, in the second week, all referrals on Tuesday etc. Patients and consultants were invited by the GPs to participate in the study.

### Measures

**Figure 1:**
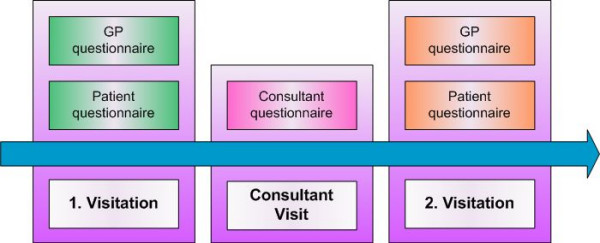
Course of questionnaire completion during the study.

GPs, consultants and patients completed short structured forms to document factual characteristics of each referral and their experiences with the referral. After the decision for a referral during a GP consultation, GPs and patients completed separately the first part of the questionnaires. The consultant received his form by the patient who visited him. He was asked by an accompanying explanational letter of the University to complete the questionnaire immediately after the consultation and send it directly via postal mailing to the University. This way was chosen to assure that the GP did not get any knowledge of the assessment by the consultant. The patient was asked to visit the GP again after the referral. After discussing the referral or results of it, GP and patient again answered part two of their questionnaires. By a referral number on each of the five questionnaires, the referral process for each patient could be followed. Figure 1 displays the course of questionnaire completion. Due to privacy reasons, this data could not be linked to personal information of the patients, as could be derived from the medical file for example. Straightforward questions were formulated to document factual referral information (specific items listed in table 1). Experiences were assessed by questions using five-point answering scales ranging from "strongly disagree", "disagree", "partly agree" to "agree" and "strongly agree" (see tables 2, 3, 4 for specific items). "Agree" was chosen as cut off, because "partly agree" was regarded as to week to represent agreement to the specific item. All items were developed for the purpose of this study. They were selected in meetings of GPs and consultants together with a research group of the University. Based on qualitative analysis of these meetings a first questionnaire was developed and tested in a small sample of referrals (30). After analysing these results, the questionnaire was reduced about 10 items to the version used in this study.

### Analysis

All analyses were done using SPSS 11.0. Frequency tables were made to describe the findings. Reliability analysis was used to develop scales for consultant experiences (4 items, alpha = 0.7151), GP experiences (5 items, alpha = 0.8058) and patient experiences (6 items, alpha = 0.7315). Items were deleted until cronbach's alpha was 0.70 or higher. Linear regression analysis (method enter) was used to determine the impact of various predictors on experiences with the referrals. The predictors of consultant experiences were consultants' medical disciplines (listed in table 1). The predictors of GP experiences were GP's purposes of the referral (4 specific items, see table 1). Predictors of patient experiences were patient age and sex, patient initiative for referral, GP initiative for referral and patient expectations (6 specific items, see table 4).

## Results

Tables 1, 2, 3 show that patients' provided data on 446 referrals, while consultants reported on 430 referrals and GPs on 411 referrals. About 60% concerned male patients, while patient age varied largely. GPs referred to various consultants, orthopedics, cardiologists, surgeons and radiologists accounted for the largest numbers. The information of who initiated the referral was collected from GPs and patients: the GP could state whom he regarded as the initiator, the patient could state if the referral was recommended by the GP or if he asked for the referral. Comparison of these data did not reveal noteworthy differences (<1%). The GPs' purposes of these referrals comprised both diagnosis and treatment. The majority of the patients was referred back to the GP.

Table 4 shows that consultants were very positive about the clarity of the referral purpose indicated by the GP (95%), the appropriateness of the referral (91%) and the information provided to the patient by the GP (87%). They were somewhat more critical regarding the information provided on the patients' medical history (61%) and prescriptions (48%). In 93 referrals (22%) consultants wanted more information on the patient. In 46 referrals (11%) they thought that the patient should have been referred earlier. Ten consultants (2%) stated that they would have preferred to refer the patient to a different medical specialist, although the GP had mentioned on the referral letter that he did not want referral to further consultants.

The experiences of consultants varied across medical disciplines. Cardiologists had less positive experiences than other consultants (beta = -0.289, p < 0.001).

A majority of the GPs had positive experiences with their referrals (Table 5). They felt that patient satisfaction with care had improved (84%), that they had received referral information timely from the consultant (77%), and were satisfied with the outcomes of the referral (77%). In 258 referrals (63%) they perceived clear diagnostic benefits, while in 202 referrals (39%) they perceived clear treatment benefits. GPs' experiences were more positive if the GP's purpose was to reduce diagnostic uncertainty (beta = 0.318, p < 0.000) or if the aim was to exclude serious illness (beta = 0.143, p < 0.010). If the GP had initiated the referral, his experiences were more positive (beta = 0.409, p < 0.001). Other purposes of the referral had no impact on their experiences.

Table 6 shows that patient expectations of the referrals mostly referred to diagnosis, including increased diagnostic certainty (80%), detailed information about the illness (66%) and exclusion of serious illness (62%). Other patient expectations referred to treatment, including recommendations for a specific treatment (59%), information about treatment options (57%) and provision of a specific treatment (53%). In a majority of the referrals, patients had positive experiences. Overall, they were satisfied with the referral (83%), felt that the consultant was well informed by the GP (81%), that the treatment in the consultants' practice was friendly (79%), and that the GP had received adequate information from the consultant (75%). Patient experiences with the referral were more positive if the initiative for the referral came from the physician (beta = 0.365, p < 0.001). Other factors which where examined had no impact on patients' experiences with the referral.

## Discussion

This study described the experiences of consultants, GPs and patients with referrals from primary care to medical specialist care. Overall, the majority of the three groups had positive experiences with the referrals. There was some variation regarding the consultants' experiences across their medical disciplines, with cardiologists having the least positive experiences. GPs (but not patients) had more positive experiences with the referral, if the referral had a diagnostic purpose. As assumed, GPs experiences were more positive if they initiated the referral. In contrast to our expectations, patients had more positive experiences if the referral had been initiated by the GP.

Previous studies revealed high and mostly unexplained variation across GPs regarding referrals [5-11]. But despite a very large variation, GPs referral rates do not correlate with the appropriateness of the referral [11]. Consequently the referral rate represents an unsatisfactory indicator of quality [12]. Our study focussed on the perceived value of the referral, including patient, GP and consultant views.

The evaluation by all involved goups, GPs, patients and specialists revealed quite good results. Some studies in other countries have shown less positive results regarding the value of referrals, but some of these studies tend to be small [8]. Studies involving larger numbers of GPs revealed different estimations of appropriateness: with 90.4% of appropriate referrals Fertig et al. found a higher rate than in the present study [7].

It could be assumed, that GPs experiences were more positive with the referral, as our study revealed. Moreover, an interesting finding of our study, which was also contrary to our expectations, was not yet assesssed nor reflected in previous studies: Patient satisfaction is associated with the initiator of the referral. We can only speculate about factors leading to the unexpected results. We assume that patients expect optimal information about disease and treatment, but they respect GPs superiority in judging if a referral is necessary or not. Our findings contradict statements of critics of the gate-keeper role, saying that patient satisfaction increases if they initiate the referral. Previous findings indicating this, were shown in completely different settings, where the patients did not have a trustfull relationship to their GP [13]. Regarding referrals, it seems that many patients may appreciate some guidance and co-ordination by the GP. GPs satisfaction was higher if the referral purpose was diagnostic than therapeutic. An explanation for this result could be that the aim of diagnostic referrals is to exclude serious diseases or to confirm suspected diseases. In most cases, the purpose is well defined and the results may help the GP to proceed in his course in case of confirmation as well as in case of exclusion. In contrast to referrals with a diagnostic aim, specialists treatments are nearly completely outside the GP's influence and may not always be perfomed according to his ideas.

The study has some limitations. Although the sample of referrals was reasonably large, it may suffer from selection bias as it was derived from a group of 25 GPs in a specific region. The study was based on self-report measures, which were not previously validated, but we believe that most questions were rather straightforward. In case of somatoform symptoms, the dissatisfaction of patients could be increased due to the fact that no organic cause has been found. It could be assumed that many of these referrals had been initiated by the patient. This could have been a source of bias, but we could only collect and link the data from consultants, GPs and patients regarding a certain referral process, but not with individual patient data. Additional information on diseases, comorbidities, social statuts, and former health care utilization would have increased the power of our data since it is known that these factors influence referrals [14]. Due to the design of the study, we have no data about the outcome of consultations that were made directly by patients without visiting a GP first. Comparing these outcomes would increase the significance of our results but is associated with multiple problems of data collection.

This observational study suggests that referrals from primary to secondary care in Germany are reasonably appropriate and that satisfaction with referrals is high among GPs, consultants and patients. Nevertheless, GPs should try to meet the different consultants' needs for different information [11]. For instance, detailed diagnostic and therapeutic information is essential for cardiologists but less important for orthopedic surgeons. Possible approaches for improvement could be frequent joint consultations or the possibility to discuss a referral with the consultant [15-18]. Sharing information on the patient, his history and medication is necessary to increase efficacy and to improve continuity of care [19]. However, the fact that GPs as well as patients' satisfaction was higher if the referral was suggested or initiated by the GP supports the role of a qualified gate-keeper.

## Competing interests

The author(s) declare that they have no competing interests.

## Authors' contributions

TR conceived and performed the study and drafted the manuscript. MW, GR, JS contributed to the study design and the manuscript. All authors read and approved the final manuscript.

## Pre-publication history

The pre-publication history for this paper can be accessed here:



## Figures and Tables

**Table 1:** Description of the referrals – Information from patients (n = 446 referrals)

Mean age of patients in years (range)	49.2 (19 -86)
Female patients	192 (43%)
Initiative for referral -Patient -GP	110 (25%) 329 (74%)

**Table 2:** Information from consultants (n = 430 referrals)

Radiology	40 (9%)
Cardiology	56 (13%)
Gastroenterology	24 (6%)
Orthopedics	91 (21%)
Urology	27 (6%)
Gyneacology	24 (6%)
Ear, nose and throat	33 (8%)
Ophmatology	23 (5%)
Surgery	42 (10%)
Other discipline	36 (8%)
Patient was known to consultant	131 (31%)
Patient was referred back to GP	387 (90%)

**Table 3:** Information from GPs (n = 411 referrals)

Reported goal of the referral:	**N (%)**
diagnosis	269 (66%)
excluding serious disease	176 (43%)
information on treatment options	111 (27%)
provision of treatment	152 (37%)

**Table 4:** Consultants' experiences (n = 430)

	N	(strongly) agree %
Clear referral goal by GP #	398	95%
Adequate information on the patients' medical history by GP #	261	61%
Adequate information on prescriptions by GP #	208	48%
More information on the patient desired	93	22%
Adequate information to patient on the referral by GP #	376	87%
I would have prefered to refer the patient	10	2%
Referral was appropriate	390	91%
Patient should have been referred earlier	46	11%

# Items that were included in the scale "consultant experiences" (alpha = 0.7151).

**Table 5:** GPs' experiences (n = 411)

	N	(strongly) agree %
I disagree with the referral	22	5%
I have received referral information timely #	318	77%
I am satisfied with the outcomes of the referral #	318	77%
The referral had clear diagnostic benefit #	258	63%
The referral had clear treatment benefit #	202	49%
Patient satisfaction improved #	345	84%

# Items that were included in the scale 'GP experiences" (alpha = 0.8058) Notes. Factor analysis suggests 1 factor without i18, that explains 61% of the variation.

**Table 6:** Patients' expectations and experiences (n = 446 referrals)

	(strongly) agree	
	
	n	%
**Expectations**		
Detailed information about my disease	294	66%
More diagnostic certainty	356	80%
Exclusion of serious illness	275	62%
Information about treatment options	255	57%
Recommendation for specific treatments	260	59%
Specific treatment is provided	238	53%
**Experiences**		
Consultant was well informed by GP #	360	81%
Friendly treatment in consultants practice #	351	79%
GP received adequate information from consultant #	333	75%
I received examinations or treatments which had not been mentioned by my GP	63	14%
I was overall satisfied with referral #	371	83%
Treatment changed due to referrals results	198	44%
Referral was unnecessary	26	6%
I received important information #	147	33%
My GP has received important information #	329	74%
My GP should have prepared me better on the referral	16	4%
Specialist should have explained me more	161	36%

# Items included in the scale "patient experiences". (alpha = 0.7315)

## References

[B1] Grumbach K, Selby JV, Damberg C, Bindman AB, Quesenberry CJ, Truman A, Uratsu C (1999). Resolving the gatekeeper conundrum: what patients value in primary care and referrals to specialists. JAMA.

[B2] Kvamme OJ, Olesen F, Samuelson M (2001). Improving the interface between primary and secondary care: a statement from the European Working Party on Quality in Family Practice (EQuiP). Qual Health Care.

[B3] Gervas J, Perez FM, Starfield BH (1994). Primary care, financing and gatekeeping in western Europe. Fam Pract.

[B4] Starfield B (1994). Primary care. Participants or gatekeepers?. Diabetes Care.

[B5] Bailey J, King N, Newton P (1994). Analysing general practitioners' referral decisions. II. Applying the analytical framework: do high and low referrers differ in factors influencing their referral decisions?. Fam Pract.

[B6] Cummins RO, Jarman B, White PM (1981). Do general practitioners have different "referral thresholds"?. Br Med J (Clin Res Ed).

[B7] Fertig A, Roland M, King H, Moore T (1993). Understanding variation in rates of referral among general practitioners: are inappropriate referrals important and would guidelines help to reduce rates?. BMJ.

[B8] Jones EG, Stott NC (1994). Avoidable referrals? Analysis of 170 consecutive referrals to secondary care. BMJ.

[B9] Moore AT, Roland MO (1989). How much variation in referral rates among general practitioners is due to chance?. BMJ.

[B10] Reynolds GA, Chitnis JG, Roland MO (1991). General practitioner outpatient referrals: do good doctors refer more patients to hospital?. BMJ.

[B11] O'Donnell CA (2000). Variation in GP referral rates: what can we learn from the literature?. Fam Pract.

[B12] Coulter A (1998). Managing demand at the interface between primary and secondary care. BMJ.

[B13] Forrest CB, Weiner JP, Fowles J, Vogeli C, Frick KD, Lemke KW, Starfield B (2001). Self-referral in point-of-service health plans. JAMA.

[B14] Allen JK, Scott LB, Stewart KJ, Young DR (2004). Disparities in women's referral to and enrollment in outpatient cardiac rehabilitation. J Gen Intern Med.

[B15] Roland M, Bewley B (1992). Boneline: evaluation of an initiative to improve communication between specialists and general practitioners. J Public Health Med.

[B16] Vlek JF, Vierhout WP, Knottnerus JA, Schmitz JJ, Winter J, Wesselingh-Megens AM, Crebolder HF (2003). A randomised controlled trial of joint consultations with general practitioners and cardiologists in primary care. Br J Gen Pract.

[B17] Forrest CB, Glade GB, Baker AE, Bocian A, von Schrader S, Starfield B (2000). Coordination of specialty referrals and physician satisfaction with referral care. Arch Pediatr Adolesc Med.

[B18] Kisely S, Horton-Hausknecht J, Miller K, Mascall C, Tait A, Wong P, Bostwick R (2002). Increased collaboration between primary care and psychiatric services. A survey of general practitioners' views and referrals. Aust Fam Physician.

[B19] Haggerty JL, Reid RJ, Freeman GK, Starfield BH, Adair CE, McKendry R (2003). Continuity of care: a multidisciplinary review. BMJ.

